# Dynamic optic nerve sheath diameter monitoring for intracranial pressure assessment in TBI patients

**DOI:** 10.3389/fneur.2026.1808654

**Published:** 2026-04-14

**Authors:** Liu Yang, Zhihui Xie, Jie Xiang, Huiting Wang, Hui Jiang

**Affiliations:** 1 Department of Emergency Medical Service, The First People’s Hospital of Changde City, Changde, Hunan, China; 2 Department of Emergency, The First People’s Hospital of Changde City, Changde, Hunan, China; 3 Medical Education Center of Jinan University, Guangzhou, Guangdong Province, China

**Keywords:** intracranial hypertension, mannitol, non-invasive, optic nerve sheath diameter, traumatic brain injury

## Abstract

**Objective:**

To explore the role of dynamic monitoring of optic nerve sheath diameter (ONSD) in the assessment of intracranial pressure (ICP) in patients with traumatic brain injury (TBI).

**Methods:**

A prospective study was conducted on patients with traumatic brain injury admitted to the Neurocritical Care Unit of the First People’s Hospital of Changde between 01/06/2024 and 30/09/2025. All enrolled patients underwent invasive ICP monitoring. A 14–5 MHz ultrasound probe was used to measure the ONSD in both eyes, with ICP values recorded simultaneously during ONSD measurements. Data were collected both before and 20 min after mannitol infusion administration. The Wilcoxon signed-rank test was used to compare ONSD and ICP before and after mannitol infusion, and Spearman correlation analysis was performed to assess their relationship.

**Results:**

A total of 71 TBI patients were enrolled in this study, including 37 males and 34 females, with a mean age of 57.1 ± 14.0 years. After mannitol infusion, ONSD significantly decreased from 6.39 (6.13, 6.73) mm to 4.94 (4.71, 5.61) mm (*p* = 0.021). Concurrently, ICP decreased from 35 (32, 41) mmHg to 21 (17, 28) mmHg, with a statistically significant difference (*p* = 0.010). Both ICP and ONSD were significantly lower after mannitol infusion compared to before infusion (*p* < 0.05). Moreover, a significant correlation was observed between ICP and ONSD both before and after infusion (Spearman’s *ρ* = 0.860, *p* < 0.01).

**Conclusion:**

Dynamic monitoring of ONSD can reflect changes in intracranial pressure in patients with TBI, demonstrating potential utility in the assessment of intracranial hypertension.

## Introduction

Traumatic brain injury (TBI) is a leading cause of morbidity and mortality worldwide, and one of the most common causes of death among individuals under 40 years of age globally (1–3). Secondary brain injuries following TBI, including intracranial hematoma and cerebral edema, can lead to increased intracranial pressure (ICP). Elevated ICP severely impairs cerebral perfusion pressure, resulting in cerebral ischemia and potentially causing brain herniation, which critically threatens patient survival (4–6). Therefore, the early diagnosis of intracranial hypertension and timely, effective reduction of ICP to improve cerebral perfusion pressure are central to the management of these patients.

Currently, invasive ICP monitoring techniques, such as external ventricular drainage (EVD), remain the gold standard for diagnosing elevated ICP. However, due to the requirement for specialized surgical expertise and a high-demand medical environment, these techniques cannot be implemented in prehospital settings or emergency departments, thereby limiting their widespread clinical application. As a representative non-invasive ICP monitoring method, ultrasound measurement of the ONSD has demonstrated favorable sensitivity and specificity in diagnosing elevated ICP (7, 8). Previous studies have confirmed a strong correlation between ONSD and invasive ICP measurements (9).

However, it remains unclear whether dynamic changes in intracranial pressure can directly influence ONSD. Therefore, this study aims to explore whether dynamic monitoring of ONSD can reflect ICP in TBI patients. If confirmed, these findings may offer preliminary insights into the potential role of ONSD monitoring in settings where invasive ICP monitoring is not readily available, and warrant further investigation into its application for ICP assessment in patients with traumatic brain injury.

## Materials and methods

### Study design

Patients with traumatic brain injury who were prospectively enrolled from 01/06/2024 to 30/09/2025 in the Neuro-Intensive Care Unit of our hospital were included in this study. The study was approved by the Medical Ethics Review Committee of Changde First People’s Hospital (Ethics Approval No. 2024-401-01), and written informed consent was obtained from the patients’ family members.

### Participant selection criteria

Patients who presented with trauma mechanisms or clinical manifestations suggestive of traumatic brain injury and a Glasgow Coma Scale (GCS) score <8 during the study period were included. All patients underwent invasive intracranial pressure monitoring upon admission to the neuro-intensive care unit of our hospital. The exclusion criteria were as follows: (1) aged <18 years; (2) optic neuropathy; (3) eye or orbit diseases; (4) history of ocular surgery; (5) undergoing decompressive craniectomy.

### General patient management

All enrolled patients received standardized neurocritical care management in the neurocritical care unit prior to baseline data collection. Mechanical ventilation was delivered using a volume-controlled or pressure-controlled mode, with tidal volume set at 6–8 mL/kg of predicted body weight, positive end-expiratory pressure (PEEP) maintained between 5 and 8 cmH₂O, and arterial oxygen saturation (SpO_2_) ≥95%. Sedation and analgesia were administered continuously using propofol and fentanyl, respectively.

### Measurement

#### Invasive ICP monitoring

Intracranial pressure was monitored continuously using invasive intraventricular ICP monitoring. This technique involves connecting an EVD to an external pressure transducer. The sensor probe is located at the tip of the drain catheter, which is positioned within the lateral ventricle—either in the frontal horn or the occipital horn. Real-time ICP monitoring is achieved via a miniature transmission line within the catheter wall, which connects to the ICP monitoring device.

#### ONSD measurement

ONSD measurements were performed by two physicians, each with 3 years of ultrasound experience. The operators were blinded to the patients’ concurrent ICP values. With the patient in the supine position and the head kept in a neutral alignment, the eyelids were gently closed and the eyes were kept as still as possible. Both eyes were protected using a single-use transparent dressing. A 14-5 MHz linear ultrasound probe (CX50, Philips, United States) was placed lightly on the closed upper eyelid without applying pressure to the eyeball.

Scans were performed in both transverse and sagittal planes. For the transverse scan, the probe was positioned horizontally on the closed upper eyelid; for the sagittal scan, it was positioned vertically. The angle was adjusted to obtain the optimal image of the optic nerve sheath on both sides. Measurements were taken at 3 mm behind the globe.

To minimize measurement error, ONSD was measured three times in each eye, resulting in a total of six measurements recorded as one set. The average of these six measurements was calculated as the subject’s ONSD value, reported to an accuracy of 0.01 mm.

#### Study protocol

We collected data from all participants, including age, gender, category of TBI (isolated brain injury/multiple trauma), intracranial lesion type, mechanism of injury, admission GCS, Injury Severity Score (ISS), systolic blood pressure (SBP), diastolic blood pressure (DBP), and mean arterial pressure (MAP) (calculated as 1/3 × SBP + 2/3 × DBP). When invasive ICP monitoring indicates ICP >20 mmHg, immediately administer a 20% mannitol infusion to reduce intracranial pressure. Osmotherapy was administered using 20% mannitol at a dose of 0.5–1.0 g/kg, infused intravenously over 10–20 min via a peripheral or central vein. Baseline measurements of ONSD, ICP, and MAP were recorded in enrolled patients before the mannitol infusion. Twenty minutes after the completion of the mannitol infusion, ONSD, ICP, and MAP measurements were taken again.

The 20-min post-mannitol assessment was selected based on pharmacodynamic, physiological, and clinical considerations. Mannitol typically begins to lower ICP within 10–20 min of administration, with peak effects occurring between 20–60 min. This timing is supported by Sakowitz et al. (10), who used a 20-min mannitol infusion in severe TBI patients and observed maximal ICP reduction at 40 min after start of infusion (i.e., 20 min post-infusion), confirming this window as physiologically relevant for capturing early treatment response. Additionally, the 20-min interval aligns with routine neurocritical care workflows, enabling consistent serial assessments without interrupting patient management.

### Statistical analysis

All analyses were performed using SPSS software (version 20.0). Categorical variables were described using frequency or percentages. For quantitative data that conformed to a normal distribution, mean and standard deviation were used for analysis; for data that did not meet the criteria for normal distribution, median with IQR were used for analysis. Spearman’s correlation analysis was performed using patient-level averaged values at baseline and after mannitol infusion. The Wilcoxon signed-rank test was used to compare differences in ONSD and ICP before and after mannitol infusion.

For each patient, the absolute changes in ONSD (ΔONSD) and ICP (ΔICP) before and after mannitol infusion were calculated as the difference between baseline and after mannitol values. Spearman correlation analysis was performed to assess the relationship between ΔONSD and ΔICP. *p* < 0.05 was considered statistically significant.

## Results

### Patient characteristics

**Figure 1:**
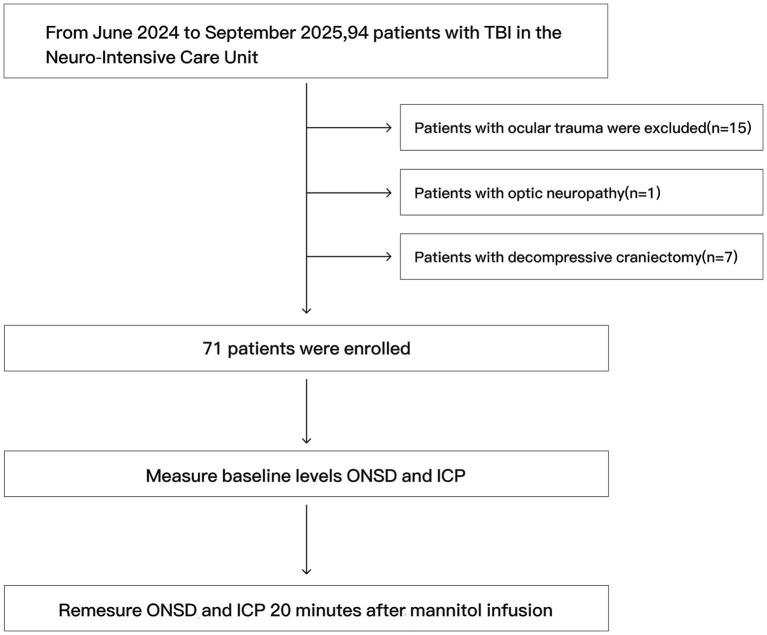
Flow chart of research.

During the study period, a total of 94 TBI patients were admitted to the neuro-intensive care unit, among whom 15 had ocular trauma, 1 had a previous history of optic neuropathy, and 7 underwent decompressive craniectomy. A total of 71 patients met the inclusion criteria for enrollment (Figure 1). The mean age was 57.1 years (SD 14.0), and 52.1% were male. Regarding intracranial lesions, acute subdural hematoma was observed in 28 patients (39.4%), traumatic subarachnoid hemorrhage in 24 patients (33.8%), and contusions in 21 patients (29.6%). Other lesion types included epidural hematoma (9 patients, 12.7%), diffuse edema (13 patients, 18.3%), and posterior fossa lesions (2 patients, 2.8%). Mixed lesions were observed in 26.8% of patients. The median GCS score was 6 (IQR 4, 7), and the median ISS was 37 (IQR 21, 58) (see Table 1).

**Table 1:** Baseline patient characteristics.

Demographics	
Age, mean (SD), years	57.1 (14.0)
Male, *n* (%)	37 (52.1)
Type of injury	
Traumatic brain injury only, *n* (%)	11 (15.5)
Multiple injury, *n* (%)	60 (84.5)
Mechanism of injury	
Motor vehicle injury	9 (12.7)
Bicycle injury	6 (8.5)
Pedestrian injury	18 (25.4)
Fall from height	33 (46.5)
Firearm or stab injury	1 (1.4)
Other	4 (5.5)
Intracranial lesion type, *n* (%)	
Acute subdural hematoma	28 (39.4)
Epidural hematoma	9 (12.7)
Contusions	21 (29.6)
Traumatic subarachnoid hemorrhage	24 (33.8)
Diffuse edema	13 (18.3)
Posterior fossa lesions	2 (2.8)
Mixed lesions	19 (26.8)
GCS score, median (IQR)	6 (4, 7)
Injury severity score, median (IQR)	37 (21, 58)
Mean arterial pressure, mean (SD), mmHg	97.1 (17.6)

### Correlation between ONSD and invasive ICP measurements

**Figure 2:**
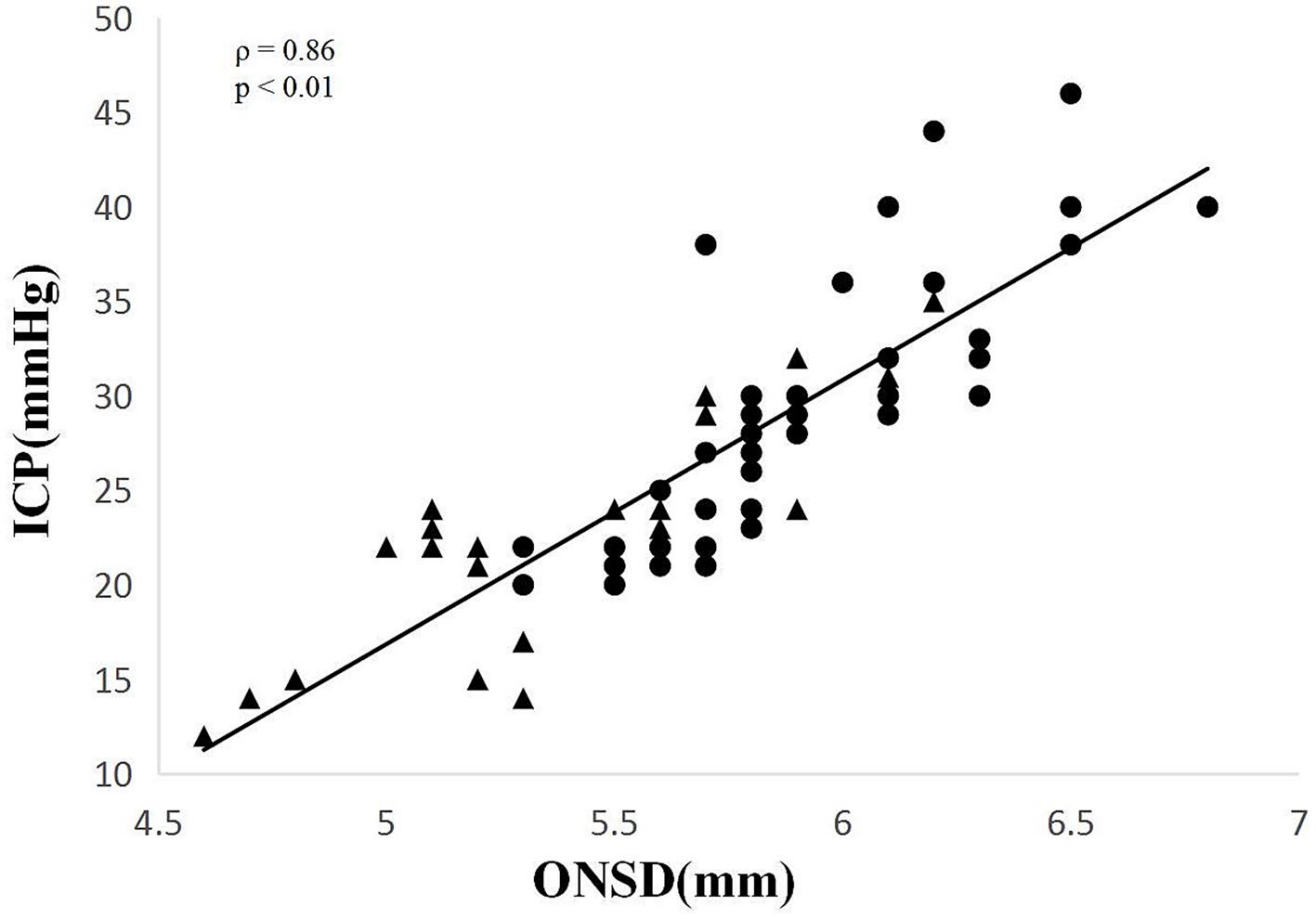
Scatter plot of ONSD versus invasive ICP monitoring before and after mannitol infusion. Black circles, baseline values; black triangles, values after mannitol infusion.

Spearman correlation analysis demonstrated a significant correlation between invasive ICP and ONSD measurements across all pooled observations (*ρ* = 0.860, *p* < 0.01) (Figure 2). Separate analyses for baseline and after mannitol measurements also showed significant correlations (Table 2).

**Table 2:** Correlation between ONSD and invasive ICP before and after mannitol infusion.

Time point	Spearman’s *ρ*	*p*-value	95% CI
Baseline	0.823	<0.01	0.712–0.895
After mannitol	0.806	<0.01	0.688–0.884

### Changes in ONSD, ICP and MAP before and after mannitol administration

As shown in Table 3, the median ONSD of enrolled patients was 6.39 (6.13, 6.73) mm before mannitol administration and decreased to 4.94 (4.71, 5.61) mm after administration. The difference in ONSD before and after mannitol use was statistically significant (*p* = 0.021). The median ICP before mannitol administration was 35 (32, 41) mmHg and decreased to 21 (17, 28) mmHg afterwards. This reduction in ICP was statistically significant (*p* = 0.010). Our study included two patients with posterior fossa hematoma. One case was a 55-year-old male with hematoma located in the cerebellar hemisphere (initial ONSD 5.7 mm, ICP 38 mmHg); the other was a 54-year-old female with hematoma located in the vermis region (initial ONSD 6.1 mm, ICP 40 mmHg). A significant correlation was observed between the two parameters before and after mannitol infusion.

**Table 3:** Changes in ONSD, ICP and MAP before and after mannitol administration.

Variable	Baseline	After mannitol	*p*
ONSD (mm)	6.39 (6.13, 6.73)	4.94 (4.71, 5.61)	0.021
ICP (mmHg)	35 (32, 41)	21 (17, 28)	0.010
MAP (mmHg)	91.1 ± 15.6	87.1 ± 9.3	0.101

**Figure 3:**
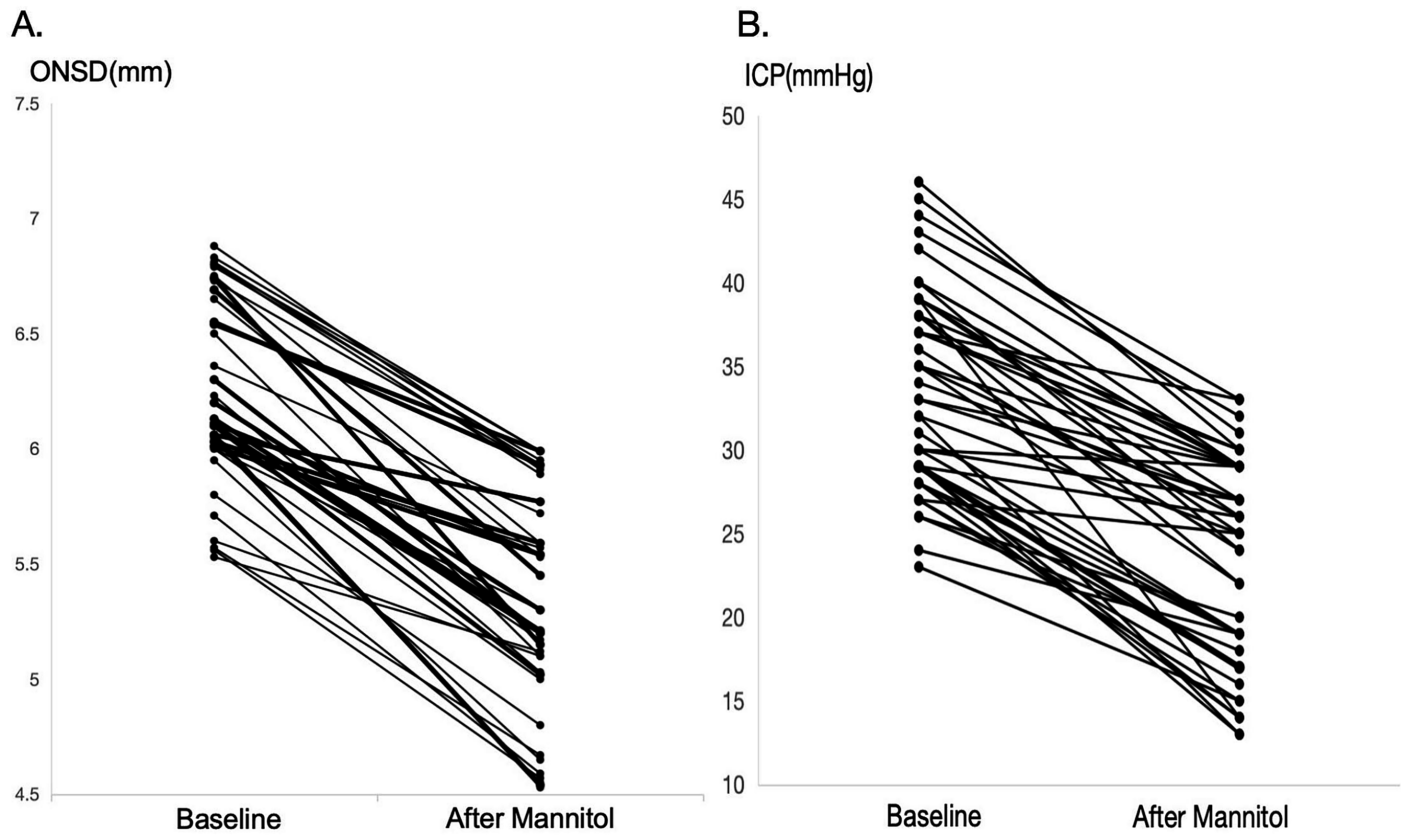
Changes in ONSD **(A)** and ICP **(B)** before and after mannitol infusion.

Figure 3 demonstrate a significant reduction in both ICP and ONSD following mannitol infusion in the enrolled patients (*p* = 0.014). The median (IQR) ΔONSD was 1.41 (1.12, 1.73) mm, and the median (IQR) ΔICP was 10.9 (7.8, 14.7) mmHg. Spearman correlation analysis revealed a significant correlation between ΔONSD and ΔICP (*ρ* = 0.78, *p* < 0.01, 95% CI: 0.66–0.86) (Table 4).

**Table 4:** Correlation between changes in ONSD and ICP after mannitol infusion.

Variable	Spearman’s *ρ*	*p*	95% CI
ΔONSD, median (IQR), mm^a^	0.78	<0.01	0.66–0.86
ΔICP, median (IQR), mmHg^b^			

a ΔONSD, the mean changes in optic nerve sheath diameter (ONSD) values measured before and after mannitol infusion.

b ΔICP, the mean changes of intracranial pressure (ICP) before and after mannitol infusion.

### Comparison of the extent of ONSD reduction across different ICP thresholds

Seventy-one patients were divided into three groups based on their pre-infusion ICP levels: <30 mmHg (*n* = 23), 30–45 mmHg (*n* = 31), and >45 mmHg (*n* = 17). The extent of ONSD reduction (ΔONSD) and ICP reduction (ΔICP) at 20 min after mannitol infusion were compared among the groups, and the results are shown in Table 5. As the pre-infusion ICP level increased, the magnitude of ICP reduction following mannitol treatment increased significantly (*p* < 0.001). In contrast, the absolute reduction in ONSD (ΔONSD) showed no significant difference among the three groups (*p* = 0.153) (see Table 5).

**Table 5:** Changes in ONSD and ICP after mannitol treatment across different ICP levels.

ICP	ΔONSD, median (IQR), mm^a^	ΔICP, median (IQR), mmHg^b^
<30 mmHg	1.25 (1.00, 1.50)	7.5 (5.0, 10.0)
30–45 mmHg	1.40 (1.10, 1.70)	11.0 (8.0, 15.0)
>45 mmHg	1.20 (0.80, 1.50)	19.0 (15.0, 24.0)
*p*	0.153	<0.001

a ΔONSD, the mean changes in optic nerve sheath diameter (ONSD) values measured before and after mannitol infusion.

b ΔICP, the mean changes of intracranial pressure (ICP) before and after mannitol infusion.

## Discussion

Our study demonstrated that for patients with TBI complicated by intracranial hypertension, after the infusion of mannitol, the ONSD significantly decreases alongside the reduction in ICP, and there is a strong correlation between the two.

Our findings suggest that ultrasound measurement of ONSD can reflect changes in intracranial pressure across different clinical states, demonstrating potential utility, particularly in settings where invasive ICP monitoring is not readily available. Raffiz et al. (8) conducted a study on 41 patients in a neurosurgical intensive care unit, finding that an ONSD of 5.2 mm served as the optimal cut-off value for identifying ICP >20 mmHg. *In vitro* experiments on human optic nerves have revealed that for every 1 mmHg increase in intracranial pressure, the ONSD expands by 0.025 mm. This finding further supports the strong correlation between ONSD values and ICP (11). However, changes in ONSD following the implementation of intracranial pressure-lowering measures in patients with elevated ICP remain unclear.

The results of this study indicate that measuring the ONSD value in TBI patients before mannitol infusion can predict their ICP, with a strong correlation observed between ONSD and ICP. These findings are consistent with those reported in prior studies (12). It is noteworthy that following the infusion of mannitol in TBI patients with elevated intracranial pressure, invasive ICP monitoring devices revealed a significant reduction in ICP levels compared to pre-infusion measurements. Concurrently, we observed a marked decrease in ONSD values as ICP declined. Spearman correlation analysis further demonstrated a strong correlation between invasive ICP measurements and ONSD values both before and after mannitol administration. The significant correlation between ΔONSD and ΔICP further supports the utility of dynamic ONSD monitoring as a non-invasive method for assessing dynamic changes in ICP. This finding directly addresses the central claim of our study that ONSD dynamics reflect ICP dynamics during osmotic therapy. A prospective study demonstrated that in pediatric patients with hydrocephalus (13), ONSD measurements significantly decreased after ventriculoperitoneal shunt surgery. This finding aligns with the results of the present study, where adult TBI patients also exhibited a declining trend in ONSD following mannitol treatment. Unlike the present study, which measured ONSD values 20 min after mannitol infusion, that research conducted ONSD measurements 30 min post-surgery. Although the methods of ICP intervention and the timing of ONSD monitoring differed between the two studies, both sets of findings demonstrated that dynamic ONSD monitoring can reflect ICP. This indicates that dynamic ONSD monitoring can reflect fluctuations in ICP over time, supporting its potential for broad clinical adoption.

The optic nerve is encased within the optic nerve sheath, and the space between the optic nerve and its sheath communicates with the subarachnoid space, allowing cerebrospinal fluid to flow freely. When ICP increases, the pressure within the sheath rises, leading to dilation of the optic nerve sheath. Therefore, theoretically, ONSD can reflect changes in ICP to some extent (14–16). Rajajee et al. (17) investigated the accuracy of ONSD in predicting elevated intracranial pressure in patients with various types of intracranial injuries (such as subarachnoid hemorrhage, traumatic brain injury, intracerebral hemorrhage, and brain tumors). The study results indicated that ONSD was the sole independent predictor of elevated intracranial pressure. This suggests that ONSD measurement holds significant predictive value for intracranial hypertension resulting from diverse pathological mechanisms. Our findings confirm that dynamic ultrasound monitoring of ONSD can reflect changes in ONSD before and after mannitol administration, as well as under different ICP levels. Moreover, these changes demonstrate a strong linear relationship with invasive ICP measurements. This demonstrates that ultrasound measurement of ONSD not only provides an estimate of ICP levels but, more importantly, enables an understanding of dynamic changes in ICP through continuous monitoring. This provides a theoretical basis for formulating appropriate intracranial pressure-lowering strategies in settings lacking invasive ICP monitoring equipment, such as prehospital environments (18).

ONSD measurement, as a non-invasive, rapid, and repeatable monitoring method with relatively low requirements for both environment and equipment, is particularly well-suited for application in resource-limited settings such as emergency departments and prehospital care. This study further validates the reliability of ONSD in intracranial pressure monitoring by assessing changes in ONSD before and after mannitol treatment. This finding not only addresses a gap in existing research but also provides clinicians with a simple and efficient tool for assessing intracranial pressure. It aids in the rapid identification of patients with elevated ICP, enabling timely intervention to reduce secondary brain injury and offering a novel solution for ICP monitoring in prehospital settings. Furthermore, the assessment of therapeutic efficacy for mannitol—a commonly used clinical agent for reducing intracranial pressure—typically relies on invasive ICP monitoring. Through ONSD measurement, this study offers a non-invasive alternative that enables dynamic monitoring of intracranial pressure changes before and after mannitol treatment. This approach not only mitigates the risk of complications associated with inappropriate mannitol use in prehospital settings—such as drastic reductions in effective circulating blood volume, renal impairment, and cerebral edema—but also equips clinicians with a means to dynamically assess therapeutic efficacy. Consequently, it supports the optimization of mannitol administration in prehospital care.

Furthermore, we observed that at extremely high ICP levels (>45 mmHg), compared to an ICP of 30 mmHg, the dynamic changes in ONSD following mannitol treatment exhibited a lagging trend. This result is considered to be related to the unique structural characteristics of the optic nerve sheath. The complex structure surrounding the optic nerve sheath is composed of collagen fibers, which enable it to bend, contract, and stretch, exhibiting elastic properties (19, 20). This serves as the anatomical basis for using ONSD to predict intracranial hypertension (21). Additionally, radial structures exist between the optic nerve and its sheath. These structures limit the expansion of the optic nerve sheath when ICP rises significantly, which explains the lag in ONSD changes observed at extremely high ICP levels (22–24). Previous studies involving intrathecal injection experiments have assessed the responsiveness of ONSD to changes in cerebrospinal fluid pressure (11). The results indicated a strong correlation between ONSD and ICP within the range of 15–30 mmHg. However, when ICP exceeds 30 mmHg, the response of ONSD to changes in ICP is delayed, which aligns with the findings of this study. This suggests that future exploration of ONSD applications in neurocritical care should take into account this structural characteristic of the optic nerve sheath.

It is worth noting that in this study, we not only observed a lag in the dilation response of ONSD to elevated ICP at extremely high ICP levels, but also found that in patients with a significant decrease in ICP after mannitol infusion, the return of ONSD to baseline levels was slower than the decline in ICP in some cases. This phenomenon may be related to the viscoelastic or hysteretic properties of the optic nerve sheath. Therefore, in clinical practice, ONSD not only exhibits a threshold effect in response to ICP elevation, but its “recovery lag” after ICP reduction also warrants attention, particularly in neurocritical care patients requiring continuous monitoring of dynamic ICP changes.

Meanwhile, this study included two cases of posterior fossa hematoma. The influence of posterior fossa hematoma on ONSD is relatively complex, as its anatomical location and pressure transmission mechanisms—potentially distinct from other types of intracranial hematomas—may lead to different effects. The posterior fossa has a relatively small volume and is compartmentalized by the dura mater, making it more susceptible to brainstem compression under pressure changes. Additionally, due to the presence of the tentorium cerebelli, elevated local pressure in the posterior fossa may not be transmitted rapidly to the supratentorial compartment where the optic nerve sheath is located. Consequently, the dilation of ONSD may be affected, particularly during the early stages of hematoma formation or when the hematoma volume is small. However, previous study indicates that the rapid response of ONSD dilation differs from the manifestation of papilledema (25), as it can occur within seconds following an increase in ICP. This rapid response makes ONSD an early indicator of acute elevations in intracranial pressure. In this study, the two patients with posterior fossa hematoma showed significant changes in both ICP and ONSD before and after mannitol infusion, with a correlation observed between the two parameters. These findings are consistent with previous research results. Therefore, this further highlights the value of dynamically monitoring ONSD changes in assessing the overall trend of ICP, which also applies to cases of posterior fossa hematoma. However, the number of posterior fossa hematoma cases in this study was limited, and there is a lack of multicenter randomized controlled trials. The accuracy of ONSD in predicting intracranial hypertension in posterior fossa hematoma requires further validation through multicenter randomized controlled studies.

In clinical practice, patients with TBI often present with concomitant thoracoabdominal injuries, which may indirectly affect ICP through various mechanisms, thereby potentially interfering with the direct correlation between ONSD and ICP. Specifically, elevated intra-abdominal pressure can increase ICP via diaphragmatic elevation and impaired venous return; increased intrathoracic pressure may inhibit superior vena cava return and elevate intracranial venous pressure. ICP elevation caused by the aforementioned mechanisms does not originate from intracranial pathology itself and may therefore lead to ONSD dilation that is inconsistent with the severity of intracranial injury, or may fail to accurately reflect dynamic fluctuations in ICP. Future studies are warranted to further explore the application value of ONSD monitoring in polytrauma patients with concomitant thoracoabdominal injuries, and to establish a stratified assessment strategy integrating multi-parameter monitoring including intra-abdominal pressure and intrathoracic pressure.

This study has certain limitations. Firstly, due to the relatively strict indications and contraindications for invasive ICP monitoring, the number of participants eligible for inclusion was limited. Although repeated measurements on the same subjects can partially mitigate the impact of small sample size on study outcomes, the potential bias introduced by a small sample cannot be overlooked. Secondly, ultrasound measurement of ONSD is influenced by the precision of the equipment, the operator’s skill level, scanning plane, technique, and interpretation. Variations in equipment and inter-observer differences may affect the study results. In this study, multiple measurements were taken bilaterally on the optic nerve sheath, and the average value was used as the final ONSD result to minimize related bias as much as possible. Finally, this study only explores the influence of intracranial pressure changes on ONSD and does not establish a precise quantitative relationship between ONSD values and ICP. More accurate results await further investigation through large-sample, multicenter prospective studies.

In summary, dynamic monitoring of ONSD can reflect the trend of ICP changes in patients with severe traumatic brain injury before and after mannitol administration, demonstrating a strong correlation between the two. This underscores the importance of dynamic ONSD measurement as a key method for assessing the presence of intracranial hypertension in TBI patients, particularly in clinical scenarios where invasive ICP monitoring is not readily available.

## Data Availability

The raw data supporting the conclusions of this article will be made available by the authors, without undue reservation.
